# Time‐dependent laryngeal desensitisation following topical lidocaine and prilocaine application in cats: a randomised controlled study

**DOI:** 10.1111/jsap.70179

**Published:** 2026-07-20

**Authors:** A. Gölgeli Bedir, L. E. Yanmaz, Y. Akçora, Ç. Özkalıpçı, Ö. T. Orhun, Y. Kocaman, S. Okur

**Affiliations:** ^1^ Department of Surgery, Faculty of Veterinary Medicine Ataturk University Erzurum Türkiye; ^2^ Department of Surgery, Faculty of Veterinary Medicine Burdur Mehmet Akif Ersoy University Burdur Türkiye; ^3^ Department of Surgery, Faculty of Veterinary Medicine Necmettin Erbakan University Konya Türkiye; ^4^ Department of Surgery, Faculty of Veterinary Medicine Yozgat Bozok University Yozgat Türkiye

## Abstract

**Objectives:**

To compare the effects of topical prilocaine and lidocaine, relative to topical 0.9% sodium chloride, on laryngeal reactivity in anaesthetised cats prior to endotracheal intubation.

**Materials and Methods:**

In this prospective, randomised, controlled, double‐blind study, 24 healthy adult cats were enrolled and allocated to topical prilocaine, topical lidocaine or topical 0.9% sodium chloride (*n* = 8/group) following anaesthesia with a standardised medetomidine‐propofol protocol. The calculated volume of each local anaesthetic corresponding to 2 mg/kg, prepared from 2% stock solutions, was diluted with 0.9% sodium chloride to a standardised final volume of 0.5 mL and atomised onto the arytenoid and vocal fold regions.

**Results:**

Relative to sodium chloride, prilocaine was associated with lower laryngeal reactivity at earlier time points (15 to 45 seconds), whereas lidocaine showed a later onset (from 60 seconds onward). Between 60 and 90 seconds, both prilocaine and lidocaine resulted in lower reactivity scores than sodium chloride, and no difference was observed between prilocaine and lidocaine at any assessed time point. No clinically evident adverse events were recorded during clinical monitoring of SpO_2_, heart rate, respiratory pattern, mucous membrane colour and clinical signs such as local irritation, hypersalivation, vomiting, marked gagging, apnoea, desaturation, bradycardia or arrhythmia.

**Clinical Significance:**

Topical prilocaine and lidocaine reduced laryngeal reactivity compared with topical 0.9% sodium chloride under the conditions of this study. The observed reductions occurred within the assessed 15 to 60 seconds interval; however, intubation conditions were not evaluated as comparative outcomes, and the clinical implications should therefore be interpreted in relation to laryngeal reactivity rather than direct measures of intubation performance.

## INTRODUCTION

Endotracheal intubation in cats must be performed quickly and safely to enable inhalation anaesthesia and to protect the airway against aspiration. However, the feline airway is small and delicate, and stimulation of the larynx may trigger reflex responses that increase intubation difficulty and the risk of airway irritation or trauma. Airway management and intubation‐related complications may contribute to perioperative morbidity and mortality in cats; therefore, techniques that facilitate atraumatic intubation are important in feline anaesthesia (Brodbelt et al., 2007; Mitchell et al., 2000). Strategies that attenuate laryngeal reflexes and facilitate atraumatic intubation, most commonly topical laryngeal desensitisation with lidocaine (LID), are routinely used in clinical practice (Dyson, 1988; Robertson et al., 2018).

Previous studies have shown that topical LID reduces laryngeal reactivity and facilitates laryngeal desensitisation in cats (Dyson, 1988; Robinson et al., 1985). Different LID administration techniques and concentrations have been evaluated, including 2% LID at approximately 2 mg/kg and 10% aerosol formulations (Dyson, 1988; Robinson et al., 1985). Although histopathological changes such as mucosal oedema and cellular injury have been reported after topical LID, these alterations were not considered clinically relevant in terms of post‐anaesthetic risk in the referenced experimental setting (Rex et al., 1983). In addition, intravenous LID does not appear to provide a clear advantage over topical delivery for suppressing laryngeal reflexes, supporting the concept that administration to the target tissue is important for clinical effect (Dyson, 1988). Plasma LID concentrations after laryngeal administration in cats have also been investigated within the studied dosing regimens in healthy cats (Soltaninejad & Vesal, 2018). More recently, Jones et al. reported that arytenoid reactivity reached its lowest level approximately 45 seconds after topical LID administration, with no severe reactions observed between 45 and 60 seconds (Jones et al., 2021). However, evidence regarding whether topical PRI provides a comparable reduction in laryngeal reactivity to LID under similar clinical conditions is limited. Therefore, direct comparison of topical PRI with LID and saline may provide clinically relevant information for laryngeal desensitisation before intubation in cats.

Prilocaine (PRI) is an amide local anaesthetic that is less commonly used than lidocaine in veterinary anaesthesia. Local anaesthetic onset, potency and duration are influenced by physicochemical properties such as pKa, lipid solubility and protein binding (Taylor & McLeod, 2020; Whiteside & Wildsmith, 2001). Although PRI has broadly comparable pharmacological characteristics to lidocaine, equipotency and the time course of effect for feline laryngeal topicalisation have not been established (Taylor & McLeod, 2020; Whiteside & Wildsmith, 2001). A clinically important safety concern for PRI is dose‐related methaemoglobinaemia; therefore, dose planning and monitoring are particularly relevant when PRI is used as a topical agent, although objective assessment requires co‐oximetry rather than reliance on mucous membrane colour or pulse oximetry alone (Garcia, 2024; Higuchi et al., 2013; Moos & Cuddeford, 2007). In cats, available evidence mainly concerns lidocaine–prilocaine combinations, such as EMLA cream, for jugular venipuncture or catheter placement and these studies should not be interpreted as direct evidence for topical PRI alone on the laryngeal mucosa (Crisi et al., 2021; Gibbon et al., 2003; Wagner et al., 2006). Therefore, this study aimed to compare topical LID, topical PRI and topical 0.9% NaCl in cats anaesthetised with a medetomidine‐propofol protocol and to evaluate laryngeal reactivity at predefined time points after topical administration. We hypothesised that topical PRI would reduce laryngeal reactivity compared with topical 0.9% NaCl and would show an effect comparable to topical LID.

## MATERIALS AND METHODS

### Study design, randomisation and blinding

This study was designed as a prospective, randomised, controlled, double‐blind trial. Cats were allocated to one of three groups using computer‐based block randomisation. An independent investigator, not involved in anaesthesia induction, laryngoscopy, intubation, scoring or data analysis, prepared the study solutions, calculated the required volumes and standardised the final administered volume. The operator performing the laryngoscopic procedure and the investigator recording outcome measures were blinded to group allocation. Following topical administration, outcomes were recorded repeatedly over a 0 to 90 seconds interval at predefined time points (T_0_, T_5_, T_10_, T_15_, T_30_, T_45_, T_60_, T_75_ and T_90_; time in seconds) (Fig S1). This study was approved by the Atatürk University Local Animal Experiments Ethics Committee (Meeting date: 28 January 2026; Meeting No. 2026/01; Decision No. 360). Written informed consent was obtained from the owners of all enrolled cats or from the relevant institution. All procedures were conducted in accordance with internationally accepted ethical principles for the use of animals in clinical research.

### Animals and eligibility criteria

A total of 24 healthy client‐owned adult female mixed‐breed domestic shorthair cats classified as ASA physical status I to II, aged 1 to 8 years and weighing 2 to 5 kg, were enrolled and allocated to three groups (*n* = 8 per group): topical LID, topical PRI and topical CON. All cats were presented for elective ovariohysterectomy and had unremarkable clinical examination findings. Preoperative screening included physical examination and routine haematological and biochemical blood tests. All cats completed the study protocol and were included in the final analysis. Exclusion criteria were respiratory disease, suspected upper airway obstruction, brachycephalic breed conformation, pregnancy or lactation, abnormalities in screening blood tests, contraindications to anaesthesia and inability to ensure safe handling due to aggressive behaviour.

### Pre‐procedural preparation

Cats were fasted for 8 hours prior to the procedure, and water was allowed until 2 hours before anaesthesia.

### Anaesthesia protocol and monitoring

All cats received medetomidine (70 μg/kg, intramuscularly into the semitendinosus/semimembranosus muscle region; Domitor®, Orion Pharma Animal Health, Finland) for premedication. Propofol was administered intravenously to effect, up to 3 mg/kg (Propofol‐PF 1%, Polifarma, Turkey). Adequate anaesthetic depth for laryngoscopy and laryngeal reactivity assessment was defined as both a marked decrease in jaw tone and loss of the palpebral reflex. Once adequate depth was achieved, direct laryngoscopy was performed and laryngeal reactivity assessments were conducted before intubation through T_90_. During this pre‐intubation assessment period, oxygen was supplied *via* face mask, and ECG, heart rate, respiratory rate, SpO_2_, mucous membrane colour and clinical respiratory pattern were monitored using a bedside physiological monitor with an integrated pulse oximetry module (C80V Veterinary Monitor, Shenzhen Comen Medical Instruments Co., Ltd., Shenzhen, China). Heart rate and rhythm were monitored by ECG, and rhythm regularity was also assessed clinically. After completion of the serial laryngeal reactivity assessments, endotracheal intubation was performed and oxygen was delivered *via* the endotracheal tube until extubation. After intubation, MAP, ETCO_2_ and rectal temperature were monitored in addition to ECG, heart rate, respiratory rate and SpO_2_. Adverse events were monitored during the pre‐intubation laryngeal assessment period and the subsequent anaesthetic period until extubation. Apnoea was defined as the absence of spontaneous respiratory movement for ≥30 seconds (Bigby et al., 2017). Respiratory depression was defined as a respiratory rate <6 breaths/minute or a clinically relevant reduction in respiratory effort requiring intervention. Bradycardia was defined as a heart rate <80 beats/minute or any decrease in heart rate requiring clinical intervention. Haemodynamic monitoring included heart rate and non‐invasive mean arterial pressure after intubation; haemodynamic instability was defined as clinically relevant bradycardia, arrhythmia or a mean arterial pressure <60 mmHg requiring intervention. Potential adverse events, including apnoea, respiratory depression, bradycardia, laryngospasm, desaturation, arrhythmia or haemodynamic instability, were recorded.

### Topical study drug administration

After visualisation of the larynx by direct laryngoscopy using a Gowllands laryngoscope set fitted with an appropriately sized curved Macintosh‐type blade (Gowllands, Croydon, UK), solutions were delivered to the bilateral arytenoid and vocal fold regions using a mucosal atomisation device (MAD; Teleflex, Morrisville, NC, USA). The LID group received 2% lidocaine (Aritmal® 2%, Osel Pharmaceutical Industry and Trade Inc., Turkey), the PRI group received 2% prilocaine (Prilacest® 2%, INCPHARMA Pharmaceutical Industry and Trade Ltd., Turkey) and the CON group received 0.9% NaCl (Polifarma® 0.9% NaCl, Polifarma Pharmaceutical Industry and Trade Inc., Turkey). For both local anaesthetics, the dose was calculated individually as 2 mg/kg using the 2% stock solution. The calculated volume of 2% lidocaine or 2% prilocaine was then diluted with sterile 0.9% NaCl to achieve a standardised final administered volume of 0.5 mL. Consequently, the final administered concentration varied between 0.8% and 2% depending on individual body weight. The same total volume (0.5 mL) of 0.9% NaCl was administered in the CON group. Timing began immediately after completion of topical administration; therefore, T_0_ represented 0 second after topical application rather than a pre‐application baseline measurement.

### Standardisation and assessment of laryngeal reactivity

To minimise variability, all laryngoscopic examinations, laryngeal stimulations and laryngeal reactivity assessments were performed by the same blinded operator using the same technique and equipment across all cats. Laryngeal stimulation was performed using the tip of a cuffed endotracheal tube of the same size (2.5 mm internal diameter; Sterimed Surgicals India Pvt. Ltd., Haryana, India) in all cats. The same 2.5‐mm ID cuffed endotracheal tube was subsequently used for endotracheal intubation in all cats after completion of the serial laryngeal reactivity assessments. At each predefined time point in seconds after topical application (T_0_, T_5_, T_10_, T_15_, T_30_, T_45_, T_60_, T_75_ and T_90_), the stimulus consisted of three consecutive gentle contacts with the midpoint of the arytenoid region using the endotracheal tube tip. The applied pressure was not quantified instrumentally; however, the stimulus was standardised by using the same operator, tube size, anatomical target, number of contacts and contact technique throughout the study. Laryngeal response was scored according to predefined criteria as follows: 0 = no visible laryngeal response; 1 = mild, brief response not interfering with tube passage; 2 = moderate response, including clear arytenoid adduction, coughing or transient complete closure lasting <3 seconds; and 3 = marked laryngospasm or complete laryngeal closure lasting ≥3 seconds. These criteria were provided to the evaluator before data collection and were applied as predefined scoring rules rather than as examples. After completion of the serial laryngeal reactivity assessments, endotracheal intubation was performed using a standardised approach by blinded personnel. Intubation‐related observations were recorded descriptively and were not analysed as comparative study outcomes. Respiratory complications during recovery were documented.

### Safety monitoring for prilocaine

Given the potential risk of PRI‐associated methaemoglobinaemia and local anaesthetic systemic toxicity, mucous membrane colour, SpO_2_, heart rate, respiratory pattern and clinical signs were closely monitored. SpO_2_ was monitored using the integrated pulse oximetry module of the bedside physiological monitor described above; co‐oximetry was not performed. Therefore, mucous membrane colour and SpO_2_ were used only for clinical monitoring and were not considered sensitive or specific enough to rule out subclinical methaemoglobinaemia. Monitored clinical signs included cyanosis or marked mucous membrane pallor, desaturation, apnoea or respiratory depression, bradycardia, arrhythmia, hypersalivation, vomiting, marked gagging, tremors, muscle twitching, seizure‐like activity or any other abnormal clinical sign suggestive of local anaesthetic toxicity. Withdrawal criteria included persistent SpO_2_ < 90%, cyanotic or markedly pale mucous membranes, severe or persistent apnoea, clinically relevant bradycardia or arrhythmia requiring intervention, neurological signs suggestive of systemic toxicity or any other adverse event judged to compromise patient safety. A supportive protocol was prepared to be implemented if clinically indicated.

### Statistical analysis

Sample size estimation was performed a priori using G*Power software (version 3.1.9.7). The calculation was based on the primary outcome variable (laryngeal reactivity score), with a significance level of *α* = 0.05 and a statistical power of 80%. The target sample size was determined to allow detection of clinically relevant differences between active treatment and control groups based on effect estimates derived from previous feline laryngeal desensitisation studies. The minimum required sample size was estimated as eight cats per group.

The primary outcome, laryngeal reactivity score (0 to 3), was an ordinal variable measured repeatedly at predefined time points (T_0_, T_5_, T_10_, T_15_, T_30_, T_45_, T_60_, T_75_ and T_90_). Therefore, laryngeal reactivity scores were summarised as median and interquartile range (Q1 to Q3). Between‐group comparisons at each time point were performed using the Kruskal–Wallis test. When a significant overall group effect was detected, pairwise *post hoc* comparisons were conducted using the Dunn–Bonferroni method. Within‐group changes over time were evaluated using the Friedman test. Following a significant Friedman test result, *post hoc* pairwise comparisons were performed between T_0_, defined as 0 second after topical application and each subsequent time point within the same treatment group, with adjustment for multiple comparisons. A *P* value <.05 was considered statistically significant.

## RESULTS

A total of 24 cats were enrolled in the study, and all 24 completed the study protocol and were included in the final analysis. No cat required additional propofol beyond the planned dose to achieve the predefined anaesthetic depth. No clinically observable adverse effects attributable to PRI or LID were observed. No clinically apparent local irritation, including visible hyperaemia or oedema, hypersalivation, vomiting or marked gagging, was recorded. During the pre‐intubation laryngeal assessment period and the subsequent anaesthetic period until extubation, no apnoea, clinically relevant respiratory depression, bradycardia, arrhythmia, desaturation or haemodynamic instability requiring intervention was recorded. No protocol modifications or early terminations occurred due to complications.

At T_0_, defined as 0 second immediately after completion of topical administration, no significant difference was detected among CON, PRI and LID (*P* = .068). Changes in laryngeal reactivity scores over time are presented in Table 1. Although the overall comparison was significant at T_5_ and T_10_ (*P* = .036 and *P* = .024, respectively), *post hoc* pairwise testing showed no significant differences for CON‐PRI, CON‐LID or PRI‐LID at either time point. At T_15_, PRI had lower laryngeal reactivity scores than CON (*P* = .026), whereas LID did not differ from CON, and PRI did not differ from LID (overall *P* = .008). At T_30_ and T_45_, PRI remained lower than CON (T_30_ *P* = .01; T_45_ *P* = .011); at these time points, LID‐CON and PRI‐LID comparisons were not significant (overall *P* = .002 and *P* = .002). From T_60_ onward, both PRI and LID showed significantly lower scores than CON, and this difference persisted at T_75_ and T_90_ (*P* < .05). No significant differences were observed between PRI and LID at any time point (Table 1).

**Table 1:** Laryngeal reactivity scores over time following topical saline, prilocaine or lidocaine in cats (*n* = 8/group)

Time (seconds)	CON Median (Q1 to Q3)	PRI^‡^ Median (Q1 to Q3)	LID^‡^ Median (Q1 to Q3)
0	3 (3 to 3)	2 (2 to 2)	2 (2 to 3)
5	2 (2 to 3)	2 (1 to 2)	2 (1 to 2)
10	2 (2 to 3)	1 (1 to 2)	2 (1 to 2)
15	2 (2 to 3)	1 (1 to 1)^**†**^	1 (1 to 2)
30	2 (2 to 2)	0 (0 to 1)^**†**^	1 (1 to 2)
45	2 (2 to 3)	0 (0 to 1)^**†**^	1 (1 to 2)
60	2 (2 to 3)	0 (0 to 1)^**†**^	1 (1 to 1)^**†**^
75	2 (2 to 3)	0 (0 to 1)^**†**^	1 (1 to 1)^**†**^
90	2 (2 to 3)	0 (0 to 0)^**†**^	1 (1 to 1)^**†**^
Within‐group *P* value	.101	<.101^‡^	.101^‡^

CON Control, PRI Prilocaine, LID Lidocaine

Data are presented as median (Q1 to Q3). Between‐group comparisons at each time point were evaluated using the Kruskal–Wallis test followed, when appropriate, by Dunn–Bonferroni‐adjusted pairwise comparisons

^†^ *P* < .05 *versus* CON at the same time point. Within‐group changes over time were assessed using the Friedman test

^‡^ Significant within‐group time effect. No significant difference was detected between PRI and LID at any assessed time point

When examined by group, laryngeal reactivity scores in CON decreased from T_0_ to T_5_ and then remained broadly consistent across subsequent time points. In PRI, scores shifted toward lower values from T_10_ onward, with a more pronounced low‐range pattern after T_30_ that persisted through T_90_. In LID, scores shifted toward lower values from T_15_ onward and showed a more consistent low‐level distribution after T_60_. For within‐group analyses, the overall effect of time was not significant in CON (Friedman *P* = .101). In contrast, the overall time effect was significant in PRI and LID (Friedman *P* < .001 and *P* = .001, respectively). T_0_‐referenced *post hoc* comparisons were interpreted cautiously because of the small sample size and adjustment for multiple comparisons (Table 1).

## DISCUSSION

The present study demonstrated that topical PRI and LID reduced laryngeal reactivity compared with the control group in anaesthetised cats. PRI showed significant reductions *versus* CON at earlier assessed time points, whereas both PRI and LID produced more pronounced suppression from T_60_ onward. No clinically evident adverse events were recorded during clinical monitoring; however, this finding should not be interpreted as definitive evidence of safety because the study was not designed or powered to evaluate safety outcomes.

In this study, the observation of low‐level desensitisation/reduced reactivity at T_0_ even in the control group, despite no topical local anaesthetic administration, suggests that laryngeal reflex responses were not determined solely by the topical agent. One plausible explanation is the standardised medetomidine‐propofol anaesthetic protocol used in all groups, which may have partially suppressed laryngeal reflexes, particularly once intubation depth was reached. Assessments were performed after propofol administration at an intubation plane defined by marked reduction in jaw tone and loss of the palpebral reflex; therefore, the low reactivity observed at T_0_ in the control group may reflect an anaesthesia‐related baseline reflex suppression independent of topical drug effects. Accordingly, the effects of PRI and LID should be interpreted not against an intact reflex, but rather as an additional desensitising contribution under the same anaesthetic conditions.

Previous evidence indicates that the timing of laryngeal desensitisation after topical lidocaine may vary depending on study conditions. Jones et al. (2021) reported that arytenoid reactivity reached its lowest level approximately 45 seconds after topical 2% lidocaine administration in cats, with no severe reactions observed between 45 and 60 seconds. In the present study, the low‐reactivity pattern in the LID group became more clearly distinguishable from the control group from T_60_ onward, suggesting that the apparent temporal pattern may vary across studies. This variability may reflect methodological and physiological differences, including application technique and mucosal distribution, standardisation of the laryngoscopic stimulus, modulation of reflex responses by anaesthetic protocol/depth, potential effects of anaesthetic drugs on local perfusion, absorption and elimination of the local anaesthetics, the analytical approach used and the relatively small sample size of the present study. In the present study, atomisation may have resulted in a different pattern of mucosal distribution compared with drop instillation. Mucosal atomisation devices have been used to deliver topical local anaesthetics to the upper airway and laryngotracheal mucosa as a fine spray during airway topicalisation procedures (Xue et al., 2007; Yadav et al., 2021). However, mucosal coverage, tissue absorption and local drug concentrations were not directly measured in the present study; therefore, any explanation related to distribution should be considered mechanistic and hypothesis‐generating rather than definitive.

Another factor that may have contributed to the observed temporal response pattern is the final concentration of the administered local anaesthetic solutions. In the present study, LID and PRI doses were standardised on a mg/kg basis, whereas the final administered volume was fixed at 0.5 mL in all cats to standardise topical delivery. Consequently, the final concentration of the local anaesthetic solution varied among cats according to body weight. Because local anaesthetic concentration can influence onset time, efficacy and duration of action, this variation may have contributed to individual differences in the onset and magnitude of laryngeal desensitisation (Becker & Reed, 2012; Lirk & Berde, 2025). However, maintaining a fixed final volume was considered important to provide a consistent topical application volume across cats while preserving the same mg/kg dose. Future studies comparing different fixed concentrations, volumes or concentration‐volume combinations are warranted to determine the optimal topical formulation for feline laryngeal desensitisation.

The shift toward lower laryngeal reactivity values in the PRI group was observed earlier than in the control group, including a lower reactivity profile at 15 seconds *versus* the control group. However, because PRI did not differ significantly from LID at any assessed time point, this finding should not be interpreted as evidence of superiority or faster onset compared with LID. This interpretation should remain clinical, as tissue‐level distribution, absorption and pharmacokinetic parameters were not directly measured. Previous studies have evaluated lidocaine–prilocaine (EMLA) formulations in cats and other species for topical procedural analgesia; however, these investigations primarily involved venipuncture, catheter placement or similar cutaneous procedures and do not provide direct evidence regarding the efficacy of topical prilocaine alone for laryngeal desensitisation before intubation (Chung et al., 2022; Crisi et al., 2021; Trinder et al., 2025; Wagner et al., 2006). Therefore, procedural context remains important, since target tissue, reflex pathways, stimulus intensity and required onset time differ substantially between cutaneous procedures and laryngeal topicalisation for intubation.

Taken together, these findings suggest that, under the conditions of this study, both LID and PRI reduced laryngeal reactivity compared with the control group; however, the clinical applicability of PRI for laryngeal topicalisation should be interpreted cautiously because direct evidence in cats remains limited. Although PRI‐LID differences did not reach statistical significance at all time points, the observed temporal pattern may still be of practical relevance in clinical situations requiring rapid intubation. PRI may be considered a reasonable alternative to LID, particularly in cases in which onset time may influence clinical decision‐making. No clinically evident adverse events were recorded during clinical monitoring in the present study. However, the study included only eight cats per group and was not designed or powered to evaluate safety outcomes. In addition, co‐oximetry was not performed; therefore, subclinical methaemoglobinaemia could not be excluded. Accordingly, the absence of clinically evident adverse events should not be interpreted as definitive evidence of safety, and the potential risk of PRI‐associated methaemoglobinaemia and other local anaesthetic‐related adverse effects, including cardiovascular, respiratory or neurological signs of systemic toxicity, should be considered in dose planning and monitoring. Further prospective studies incorporating objective methaemoglobin assessment, larger sample sizes and structured safety evaluation are needed before robust safety recommendations can be made.

This study has several limitations. First, the primary endpoint was the laryngeal reactivity score, which, although clinically meaningful, is an observer‐based ordinal measure; tissue‐level drug distribution/absorption and pharmacokinetic parameters were not directly assessed. Therefore, interpretations regarding a potentially earlier temporal effect of PRI are based on the observed clinical score pattern. Second, the study population consisted of healthy cats (ASA I to II; 1 to 8 years; 2 to 5 kg) evaluated under a medetomidine‐propofol protocol, which limits generalizability to cats with upper airway disease, brachycephalic cats or higher‐risk/critical patients. The anaesthetic protocol and intubation depth may also have influenced reflex responses and contributed to the temporal profile observed. Third, although clinical monitoring for methaemoglobinaemia was performed, laboratory confirmation (*e.g*. co‐oximetry/methaemoglobin measurement) was not undertaken; thus, subclinical changes cannot be excluded. Other local anaesthetic‐associated adverse effects were monitored clinically, but the study was not designed or powered to evaluate safety outcomes. Fourth, intubation‐related observations were collected after completion of the 0 to 90 seconds serial reactivity assessments and were recorded descriptively rather than analysed as comparative outcomes; therefore, the findings should not be interpreted as evidence of improved intubation performance. Finally, the small sample size, ordinal scoring, repeated measurements and adjustment for multiple comparisons limited the ability to demonstrate statistically significant within‐group *post hoc* differences at individual time points. The study was powered primarily to detect differences between active treatment and control groups rather than to demonstrate equivalence or non‐inferiority between LID and PRI. Consequently, the absence of statistically significant differences between the two active treatments should not be interpreted as definitive evidence of equivalent efficacy. Because repeated measurements were analysed at individual time points rather than within a mixed‐effects framework, within‐subject correlations were not explicitly modelled. Therefore, temporal within‐group findings should be interpreted cautiously and considered exploratory.

In conclusion, topical PRI and LID reduced laryngeal reactivity compared with the control group in anaesthetised cats. PRI showed a potentially earlier temporal effect pattern at some early time points, and both agents produced more pronounced suppression from T_60_ onward. No statistically significant difference was detected between PRI and LID at any time point; therefore, these findings should not be interpreted as evidence of superiority of either active treatment. Because intubation performance outcomes were not evaluated, the clinical implications should be limited to laryngeal reactivity under the conditions of this study. The absence of clinically evident treatment‐related adverse events in this small sample suggests short‐term clinical tolerability, but it should not be interpreted as definitive evidence of safety. Larger prospective studies incorporating objective methaemoglobin assessment, structured evaluation of local anaesthetic‐related adverse effects, dose optimisation and different clinical patient populations are needed.

## Author contributions

**L. E. Yanmaz:** Writing – review and editing; supervision; conceptualization. **Y. Kocaman:** Visualization; resources; investigation; writing – review and editing. **S. Okur:** Formal analysis; writing – original draft; writing – review and editing. **Y. Akçora:** Methodology; investigation; data curation; writing – review and editing. **Ç. Özkalıpçı:** Validation; resources; methodology. **Ö. T. Orhun:** Methodology; resources; supervision; software. **A. Gölgeli Bedir:** Writing – original draft; writing – review and editing.

## Conflict of interest

No conflicts of interest have been declared.

## Funding

No specific grant/equipment/drug support was received.

## AI disclosure statement

During the preparation of this manuscript, artificial intelligence‐based language tools were used only for language editing, grammar checking and translation support. These tools were not used for study design, data collection, data analysis, interpretation of results or generation of scientific conclusions. All AI‐assisted text was reviewed, edited and approved by the authors, who take full responsibility for the content, accuracy and integrity of the manuscript.

## Supporting information


**Fig S1.** Graphical summary of study design, experimental timeline and laryngeal reactivity outcomes following topical laryngeal desensitisation in cats.

## Data Availability

The data that support the findings of this study are available on request from the corresponding author. The data are not publicly available due to privacy or ethical restrictions.
