# Supplementary material for: Time‐dependent laryngeal desensitisation following topical lidocaine and prilocaine application in cats: a randomised controlled study
Source: J Small Anim Pract. 2026 Jul 20;67(9):836–42. doi: 10.1111/jsap.70179 (PMC13539693; doi:10.1111/jsap.70179)
Supplement: Supplementary file 1 [file JSAP-67-836-s001.docx]

**Supplementary Figure S1**. Graphical summary of study design, experimental timeline, and laryngeal reactivity outcomes following topical laryngeal desensitization in cats.

**Abbreviations:** ASA, American Society of Anesthesiologists physical status; CON, control; LID, lidocaine; PRI, prilocaine; NaCl, sodium chloride; s, seconds.
